# Topography dependence of the metamagnetic phase transition in FeRh thin films

**DOI:** 10.1038/s41598-020-60767-z

**Published:** 2020-03-04

**Authors:** J. L. Warren, C. W. Barton, C. Bull, T. Thomson

**Affiliations:** NEST Research Group, School of Computer Science, The University of Manchester, Oxford Road, Manchester, M13 9PL UK

**Keywords:** Magnetic properties and materials, Nanowires, Ferromagnetism, Magnetic properties and materials, Information storage

## Abstract

The equiatomic alloy FeRh is of great scientific and technological interest due its highly unusual first-order antiferromagnetic (AF) to ferromagnetic (FM) phase transition. Here we report an exploration of the interplay between topography and phase evolution with a comprehensive magnetic force microscopy study of nominal 50 nm thick FeRh thin films and subtractively patterned wires of width 0.2 µm–2 µm. In continuous films where the surface morphology had not been optimised for smoothness, the topographical variation was observed to dominate the distribution of the magnetic transition temperatures and dictates the nucleation and growth of the magnetic phases. This observation was repeated for patterned elements, where the effects of surface morphology were more significant than those of spatial confinement. These results have clear implications for future studies of low-dimensional FeRh films, as surface topography must be considered when analysing and comparing the transition behaviour of FeRh thin films.

## Introduction

In the 1930’s Fallot and Hocart^1,2^ reported a sharp increase in the magnetisation of bulk samples of the equiatomic alloy FeRh under heating through a critical temperature, T_r_ ≈ 370 K. A temperature hysteresis of ≈ 10 K associated with the magnetisation change suggested the transition was first-order in nature. Interest in FeRh was revived in the 1960s, when Muldawer and de Bergevin^3^ found that the magnetisation change corresponded to a transition from an antiferromagnetic (AF) phase to a ferromagnetic (FM) phase, and Kouvel and Hartelius^4^ reported a reduction in resistivity upon heating. Since these studies, it has also been shown that the transition can be induced through the application of a variety of additional external stimuli, such as pressure^5^, applied magnetic^6,7^ and electric fields^8^, and spin polarised currents^9^. Recent studies^10–12^ have reported ultrafast generation of FM order in FeRh under heating from femtosecond laser pulses. This opens the possibility of multifunctional switchable spintronic devices modulated through subpicosecond onset of the transition under laser irradiation, with operating frequencies far beyond those currently achievable using conventional electronics. In order to realise these devices and maximise their efficiencies a fundamental understanding of the transition in technologically relevant thin films and patterned nanoscale wires is vital.

The transition temperature has been shown to be strain-tuneable^5,13–15^, and therefore the slight strain inhomogeneities present in epitaxially grown FeRh films are expected to produce local variations in transition behaviour. The progression of the transition and the intermixing of the two magnetic phases over the transition region was imaged directly by Baldasseroni *et al*. via X-ray magnetic circular^16^ and linear^17^ dichroism in photoemission electron microscopy. They reported an asymmetry in domain evolution across the heating and cooling transitions. Upon heating they observed heterogeneous nucleation of the FM phase at different sites followed by domain growth, whereas upon cooling the formation of the AF phase was dominated by nucleation at defects, with little subsequent growth. These findings were supported in later work by Uhlir *et al*.^18^ who found the asymmetry in the transition was strongly enhanced when FeRh was patterned into mesoscale stripes.

Strain variation in thin films can impact growth and subsequently produce regions of differing surface morphology. As a result, in FeRh thin films a strong link between topography and phase evolution is expected. In this work a systematic magnetic force microscopy (MFM) study of FeRh thin films and nanowires is presented to explore this hypothesis. Variable temperature MFM has been shown in previous studies^19–22^ to be a valuable tool when direct imaging of the FeRh phases is required. However, a detailed MFM study of FeRh films has not previously been reported with the majority of studies confined to temperatures at or around T_r_. Furthermore, few studies have investigated the effects of laterally confining FeRh films where MFM offers a resolution of a few 10’s of nm and direct imaging using MFM of the transition in patterned structures has also not been reported previously. The goal of this work is two-fold: to directly image the evolution of the FM and AF phases across the heating and cooling transitions, and to compare the results obtained for continuous films and patterned FeRh nanowires to investigate the impact of spatial confinement with a resolution in the 10’s of nm regime.

## Film Characterisation

**Figure 1:**
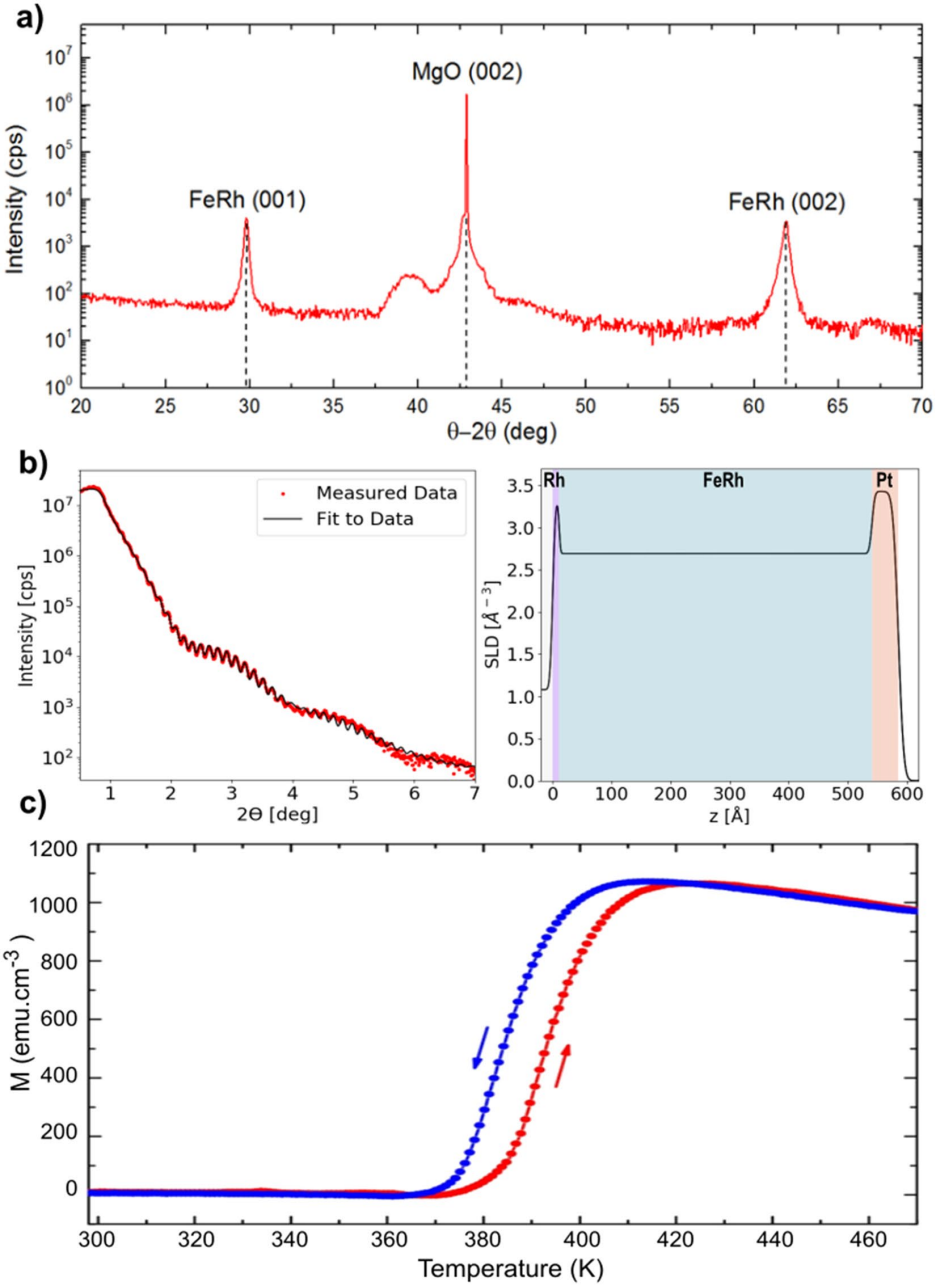
(**a**) XRD data for an MgO(001)/Rh(1.1 nm)/FeRh(53 nm)/Pt(4.5 nm) showing the FeRh(001) superlattice and FeRh(002) fundamental peaks. (**b**) Fitted XRR data (left) with electron scattering length density depth profile (right). (**c**) VSM data showing that the FM phase started to developed at ≈ 370 K and grew as the temperature was increased further, producing a smooth increase in moment reaching a maximum at ≈ 420 K. In the cooling branch, the reverse transition began at ≈ 405 K, producing a gradual drop in the moment until - at ≈ 360 K - the cooling transition was complete. The two transitions are identical in shape, exhibiting a thermal hysteresis of ≈ 10 K.

Nominal 50 nm thick films of Fe_50_Rh_50_ were sputter-deposited onto single-crystal MgO(001) substrates including a capping layer of Pt 4 nm to inhibit oxidation. The films were not optimised for metallic smoothness. As the metamagnetic phase transition is only observed in the α”-FeRh phase, X-ray diffraction (XRD) measurements were performed (Fig. 1a) to confirm the correct crystal structure had been achieved. The perpendicular lattice constant was calculated from the position of the superlattice peak (001) as ≈ 2.9956 Å. Comparison with the bulk lattice parameter ≈ 2.9978 Å gave a value of the compressive strain of ≈ 0.22%, indicating good epitaxial growth and low lattice mismatch. X-ray reflectivity (XRR) analysis is shown in Fig. 1b was undertaken to investigate the depth dependent structure of the films. The best fit to the data was obtained by including a thin Rh rich layer adjacent to the MgO substrate, as has been reported previously^23^. The FeRh thickness was determined to be 53 nm with a roughness of 0.4 nm close to the intended film thickness, yielding a film structure of MgO(001)/Rh(1.1 nm)/FeRh(53 nm)/Pt(4.5 nm). Variable temperature vibrating sample magnetometry (VSM) demonstrated that these films exhibit an AFM to FM phase transition at the expected temperature (Fig. 1c) confirming that films with the correct magnetic properties had been grown successfully.

## Topographical and MFM Analysis of FeRh Continuous-Films

**Figure 2:**
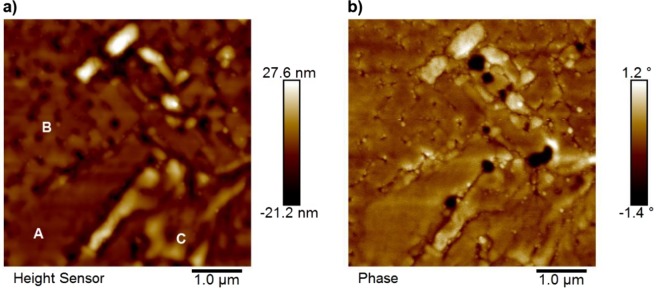
(**a**) Topological scan of the surface of an MgO(001)/Rh(1.1 nm)/FeRh(53 nm)/Pt(4.5 nm) film. The three types of surface topography are labelled. (**b**) Room temperature FM phase contrast at the same location. A clear link between surface morphology and residual phase was observed, as the location of magnetic signal coincided with the interiors or boundaries of regions of C-type structure. This scan was performed with a drive amplitude of 35 mV and a lift height of 40 nm.

Atomic force microscopy (AFM) of continuous films (Fig. 2a) showed significant variation in surface topography. Three distinct morphologies were observed and identified. The first - labelled A - was a relative large, flat expanse of uniform film. The second - labelled B - comprised of a series of ≈ 0.50 µm × 0.35 µm grains separated by troughs and holes. The third - labelled C - comprised of rounded bulbous stripes of material which increased the maximum variation in film height to ≈ ±25 nm, a value which was not representative of the majority of the film surface as demonstrated by the XRR roughness data.

These surface types were observed across the entire film at every location scanned, and were common to all films studied. It can be observed that the boundaries between grains met at 45° to the substrate axes, which are approximately horizontal and vertical in Fig. 2a as expected for the growth of FeRh on MgO where the FeRh B2 [110] in-plane direction is rotated by 45° relative to the MgO cubic [200] direction^24^. This suggests that a twinning effect resulting from imperfections in the underlying substrate may be responsible for the growth of C-type regions through the creation of a twin boundary which propagates at an angle relative to the film normal.

Room temperature (293 K) MFM studies (Fig. 2b) revealed the presence of pockets of residual FM phase throughout the film. This observation is in contrast to previous studies by Baldasseroni^25^ who observed no room-temperature interfacial ferromagnetism in Pt-capped FeRh films. Comparison of the height and phase data showed there was a strong correlation between locations of the FM phase and topological features, as magnetic signal was observed in or at the boundaries of regions exhibiting C-type structure. This observation is suggestive of a clear link between magnetic phase and morphology.

**Figure 3:**
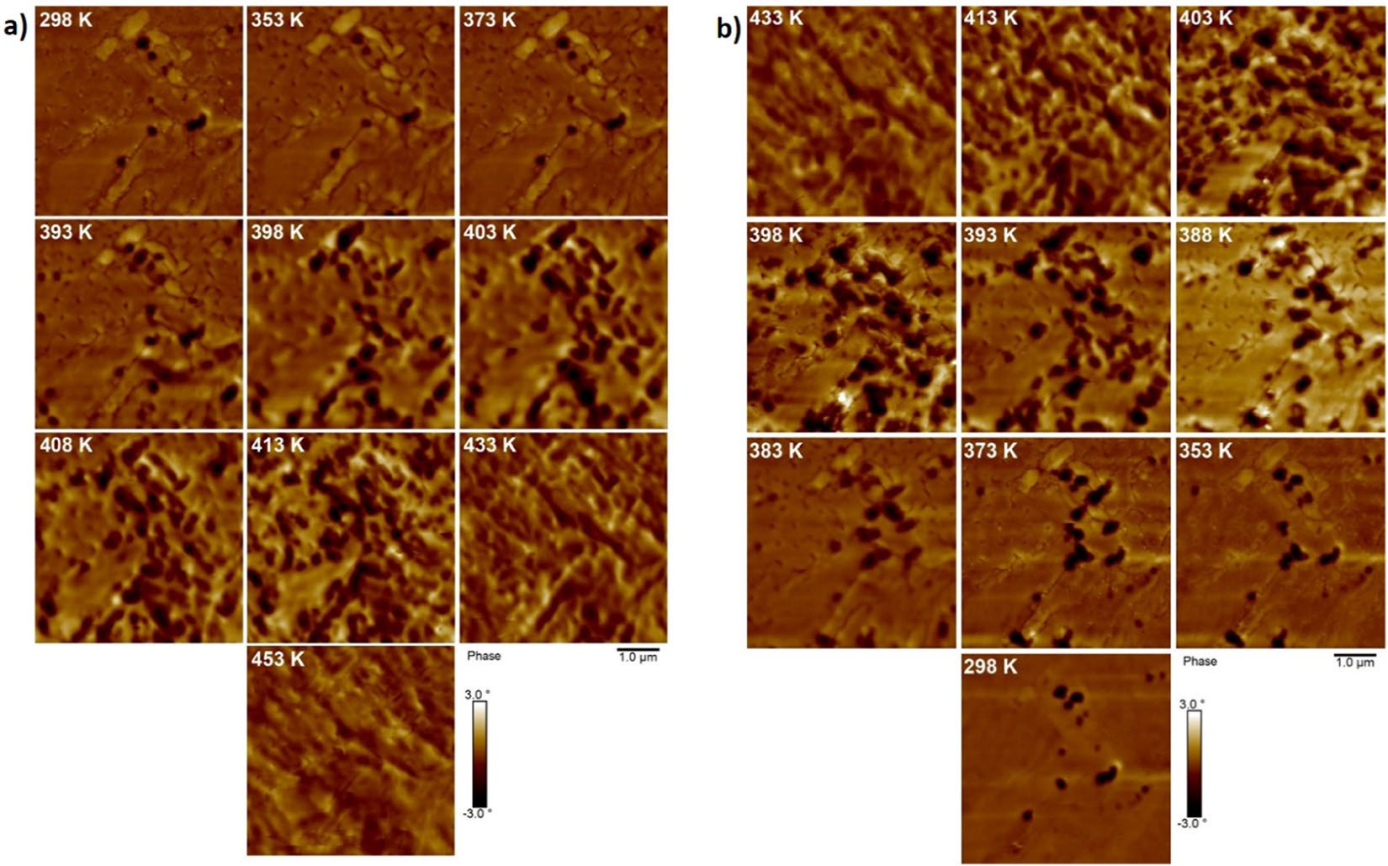
MFM scans showing the (**a**) heating and (**b**) cooling transitions in a MgO(001)/Rh(1.1 nm)/FeRh(53 nm)/Pt(4.5 nm) film. All scans have been normalised to the same phase range to aid comparison. The temperature at which the scans were performed is indicated. The scans were performed with a lift height of 40 nm and drive amplitudes in the range 12 mV–44 mV.

Under heating (Fig. 3a) the FM phase remained unaltered until new domains nucleated at 393 K. As previously reported^16–18^ at low temperatures the evolution of the FM phase was nucleation-driven, with limited growth of existing domains. Regions of FM phase were approximately circular in shape, with lateral areas in the range 0.1 µm^2^–0.55 µm^2^. Above 393 K this trend reversed, as the growth of existing domains replaced nucleation as the dominant evolution method. This growth continued until - at 433 K - the entire film was in the FM phase. Raising the temperature further led to domain coalescence, which continued until 453 K. These results were consistent with previous studies of FM phase evolution using photoemission electron microscopy (PEEM) reported by Baldasseroni *et al*.^16^.

During the reverse cooling transition (Fig. 3b) the ferromagnetic domains began to separate, reversing the coalescence observed at the end of the heating transition. At 413 K, the AF phase nucleated in two small distinct regions which grew rapidly as the temperature was lowered, such that between 403 K and 398 K a large portion of the film transitioned to the AF phase. This indicated that evolution of the AF phase was driven by the growth of pre-existing domains rather than nucleation of new domains, in contrast to behaviour reported in previous studies^16–18^ where domain growth was minimal. A significant amount of FM phase remained at 353 K and when the sample returned to the starting temperature of 298 K the initial magnetic signal pattern was reproduced.

**Figure 4:**
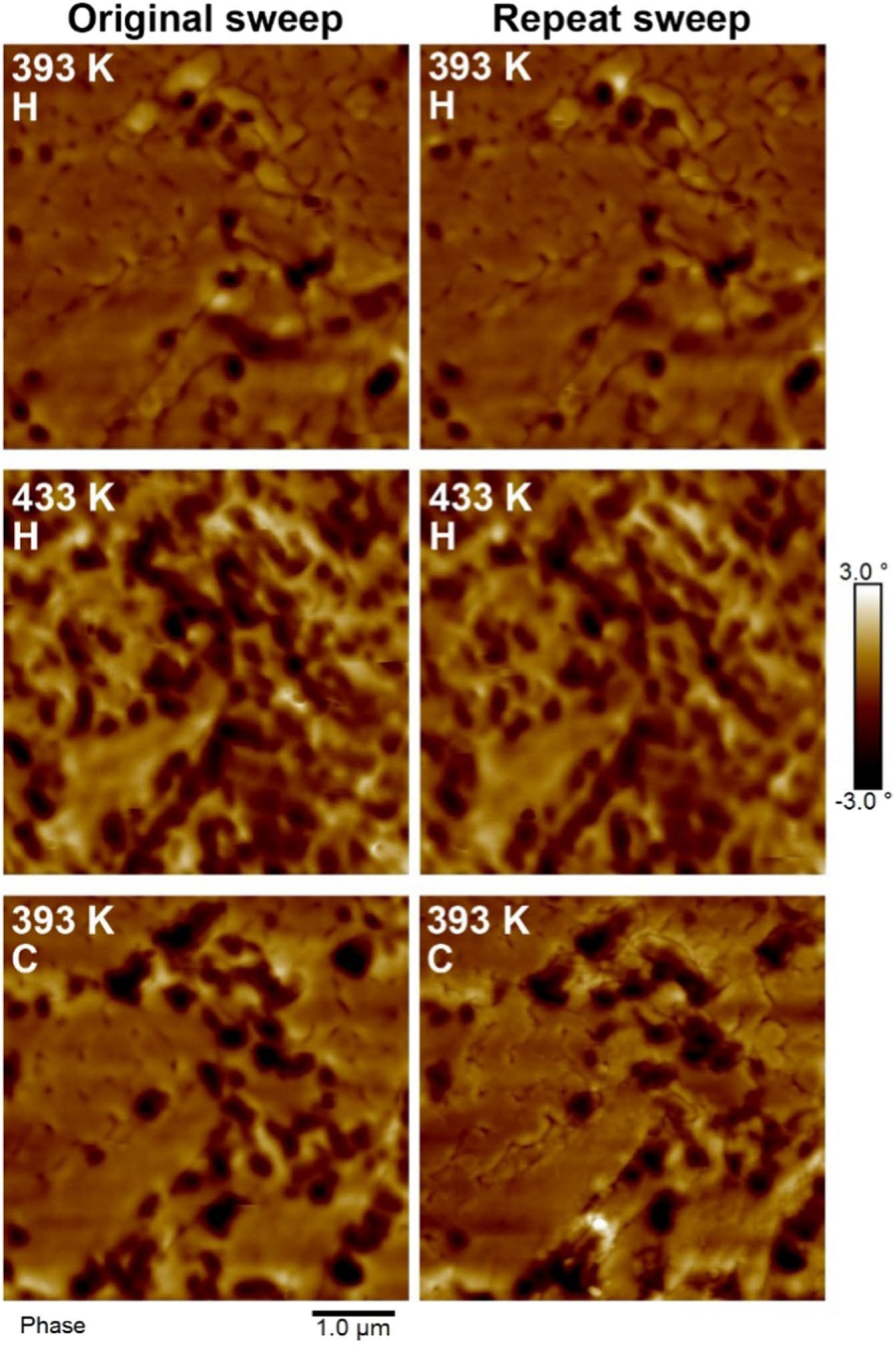
MFM scans showing the repeatability of the transition in an MgO(001)/Rh(1.1 nm)/FeRh(53 nm)/Pt(4.5 nm) film. All scans have been normalised to the same phase range to aid comparison. The numbers indicate the temperatures at which the scans were performed, with “H” and “C” indicating whether they were part of the heating or cooling transition respectively.

The heating and cooling transitions were repeated to test the reproducibility of the phase evolution as shown in Fig. 4. These data demonstrated that at the same points in successive sweeps the domain structure was almost identical, mirroring the reproducibility of the residual FM phase. This indicates that the residual phase is directly linked to the local film morphology.

**Figure 5:**
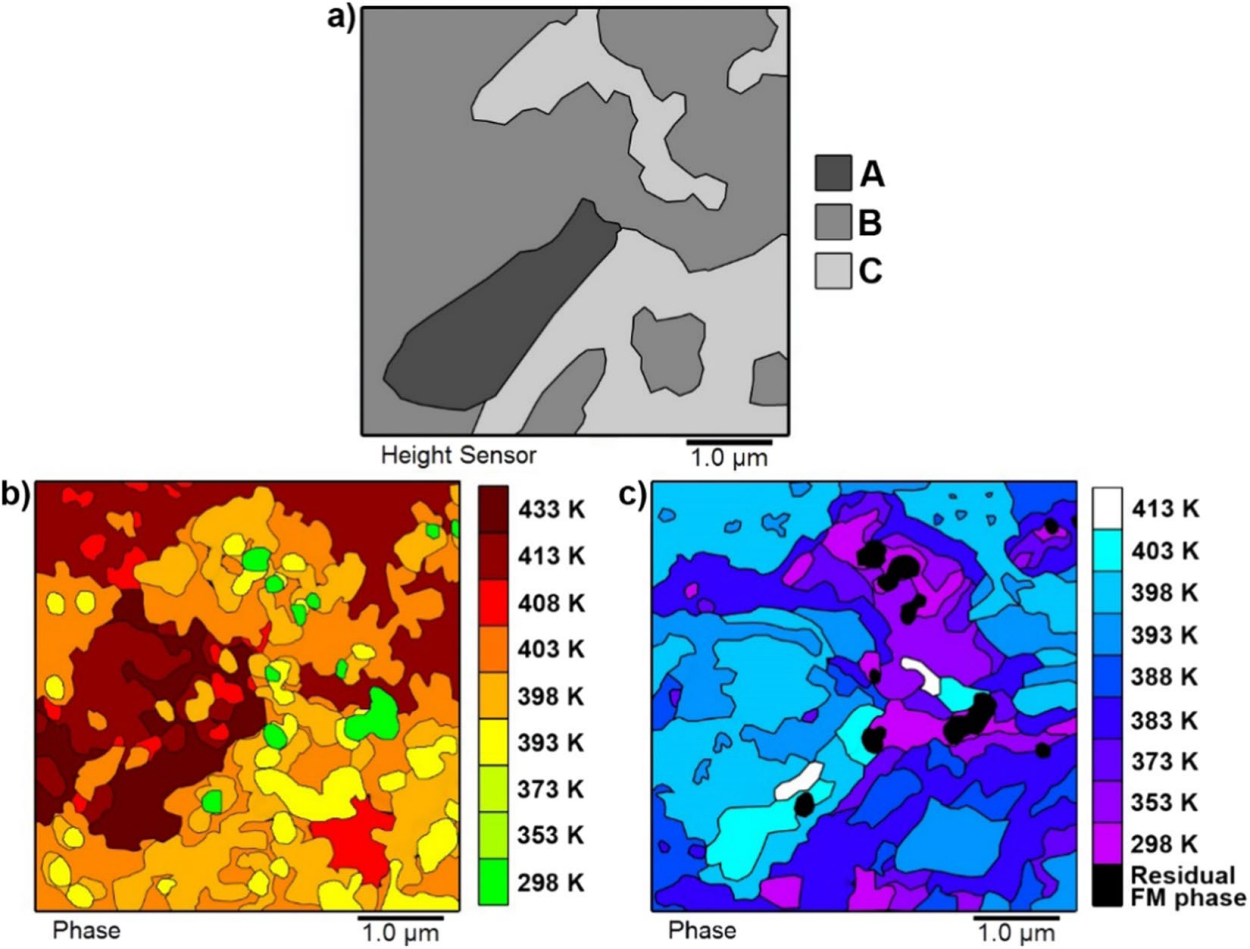
Heat maps representing the evolution of the heating and cooling transitions in a MgO(001)/Rh(1.1 nm)/FeRh(53 nm)/Pt(4.5 nm) film. (**a**) A topography map showing the distribution of A-type, B-type and C-type surface structures. (**b**) The evolution of the FM phase during the heated transition. The key refers to the temperature at which FM order first appeared in the phase data. (**c**) The evolution of the AF phase during the cooling transition. The key refers to the temperature at which AF order first appeared in the phase data. The areas at which residual FM phase was present at room temperature are coloured black.

In order to explore the link between structure and phase evolution “heat maps” were produced which plotted the variation of the heating and cooling transition temperature across the scan region, Fig. 5. A comparison of Fig. 5a,b shows a clear link between transition temperature and local microstructure. Regions containing B-type topography transitioned at a temperature approximately 10 K higher than those with C-type structure, with A-type regions transitioning approximately 10 K higher than the B-type regions. These results provide evidence for a model where domains nucleate in C-type regions before growing first into B-type then A-type areas as the temperature increases. This resulted in rapid domain growth at higher temperatures, as large A-type and B-type regions transitioned effectively as a single step – shown by the large, uninterrupted dark red areas in Fig. 5b. Such a specific link between film morphology and transition temperature has not previously been reported and demonstrates that the importance of FeRh surface structure types and their impact on the phase transition.

A comparison of Fig. 5b,c clearly demonstrates a hysteresis of ≈ 10 K–15 K between the heating and cooling transitions - in agreement with macroscopic VSM data. Interestingly, it was observed that the hysteresis remained approximately constant across the film. For example, the majority of the B-type regions had a heating transition temperature of 413 K and a cooling transition temperature of 398 K. This indicated that different regions underwent discrete temperature hysteresis cycles that were offset with respect to each other with a clear hierarchy of phase transition temperatures T_r_ C < T_r_ B < T_r_ A.

**Figure 6:**
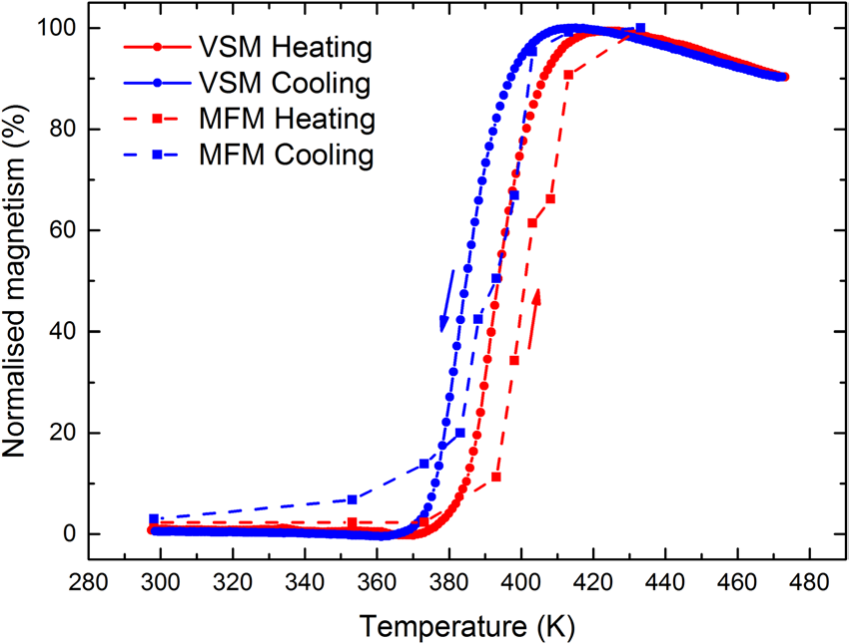
Comparison of VSM and MFM data for the MgO(001)/Rh(1.1 nm)/FeRh(53 nm)/Pt(4.5 nm) film. The MFM data were extracted from heat maps showing the percentage area of film in the FM phase during heating and cooling cycles. The VSM data are included for comparison and plotted on the same percentage scale. The dashed lines show a smoothed spline between data points, and are included solely as indication of the data trend.

Using the heat maps it is possible to extract the percentage of the scan area (5 μm^2^) in the FM phase at each point in the temperature cycle. These data can be used to estimate the percentage of the area in the FM phase. Figure 6 shows a comparison between the FM phase measured using MFM and that measured using VSM (8 mm diameter sample). The two sets of data are in very good agreement with an identical temperature hysteresis of 10 K and a similar Tr = 397 K (heating). There is a systematic offset of 8 K between the data sets which we ascribe to uncertainty in the temperature measurement most likely due to the calibration of the MFM temperature stage.

## Topological and MFM Analysis of FeRh Nanowires

As noted in previous studies^18^ when laterally confining FeRh, the AF and FM correlation lengths must be considered. The AF correlation length is limited by local structure disorder to ≈ 0.3 µm^16,17^, such that in structures ≤ 0.3 µm domains have little correlation and switch independently of one another. Conversely, FM correlations are robust to local disorder with a characteristic length of ≈ 0.55 µm^16^, stabilising the FM phase in structures ≤ 0.5 µm and shifting the cooling transition to a significantly lower temperature than observed in continuous films^18^. In order to investigate the impact of spatial confinement on the observed interplay between topography and phase transition a series of patterned FeRh nanowire devices were fabricated using a subtractive process (see methods). Wire widths in the range 2 µm–0.2 µm were investigated. A 2 µm wire was chosen as it was expected to be outside the range of magnetic correlation effects and therefore exhibit a continuous-film-like transition. This wire was intended to act as a control sample highlighting any changes in transition behaviour resulting from the fabrication procedure, for example due to etch damage or edge effects. Widths of 0.75 µm, 0.50 µm and 0.35 µm were chosen to investigate variation in the transition with critical dimensions around the expected correlation lengths. Finally, a 0.20 µm wire was fabricated to investigate the effect on the heating transition of patterning a structure with a width below that of the expected AF correlation length.

Topological analysis showed the patterned nanowires were continuous, of constant width and exhibited no obvious damage from the fabrication process. Wire widths were found to be ≈ 5–10% narrower than intended due to a higher-than-expected ion-mill rate. As with the continuous film, regions of different surface morphology were observed.

All wires were fabricated from the same FeRh film which ensured consistency across all the wires studied. The same region on all wires - the centre - was imaged. This choice removed any bias from the imaging process which might have arisen from the observed surface structure or the presence or lack of residual FM phase.

**Figure 7:**
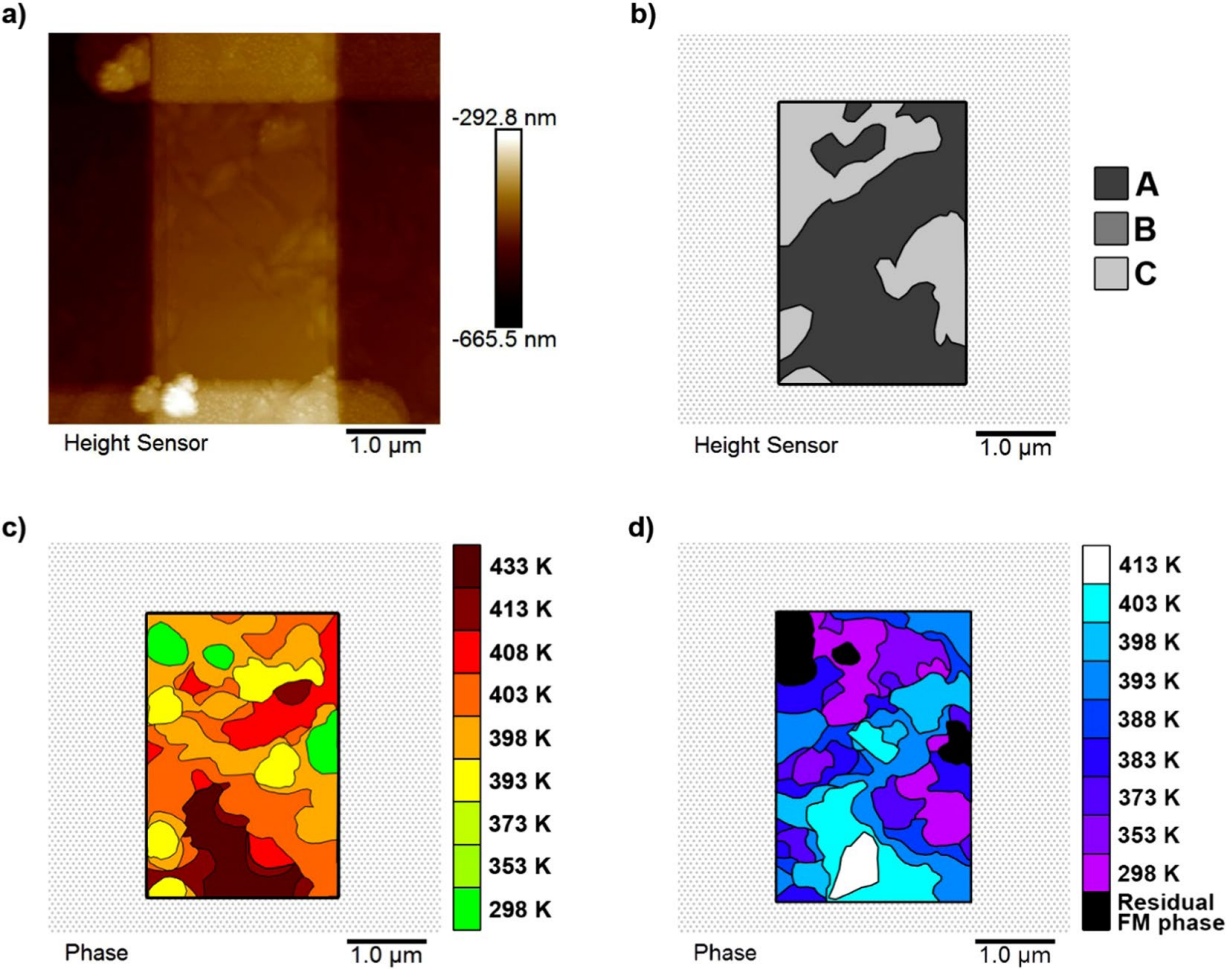
The raw and extracted data from the 2 μm wide MgO(001)/Rh(1.1 nm)/FeRh(53 nm)/Pt(4.5 nm) nanowire. (**a**) An AFM image showing the measured height data. (**b**) A topography map detailing the surface structure types observed in the wire, and their locations on the wire surface. (**c**) Heat, and (**d**) cool maps showing the evolution of the FM phase throughout a temperature sweep. All data are plotted on the same scale to aid direct comparison.

The 2 µm wire shown in Fig. 7 exhibited similar behaviour to that of the continuous film with the local surface structure, Fig. 7a, characterised into the same topography types as the continuous films, Fig. 7b, dominating the transition. The heating transition, Fig. 7c, progressed through initial domain nucleation over a range of temperatures in the C-type topography, before rapid domain growth into the A-type regions at high temperatures. The cooling transition, Fig. 7d, started with nucleation of a single AF domain in the centre of the A-type topography, before growing to fill the remaining A-type regions, and eventually the C-type regions at a significantly lower temperature. Once cooled back to room temperature the initial residual FM phase pattern was reproduced. As with the continuous film, the C-type regions favoured the FM phase and resisted AF phase, while A-type phase resisted FM phase and favoured AF phase. A hysteresis of 10 K–15 K was clearly visible, as neighbouring regions underwent separate, offset, temperature hysteresis loops.

**Figure 8:**
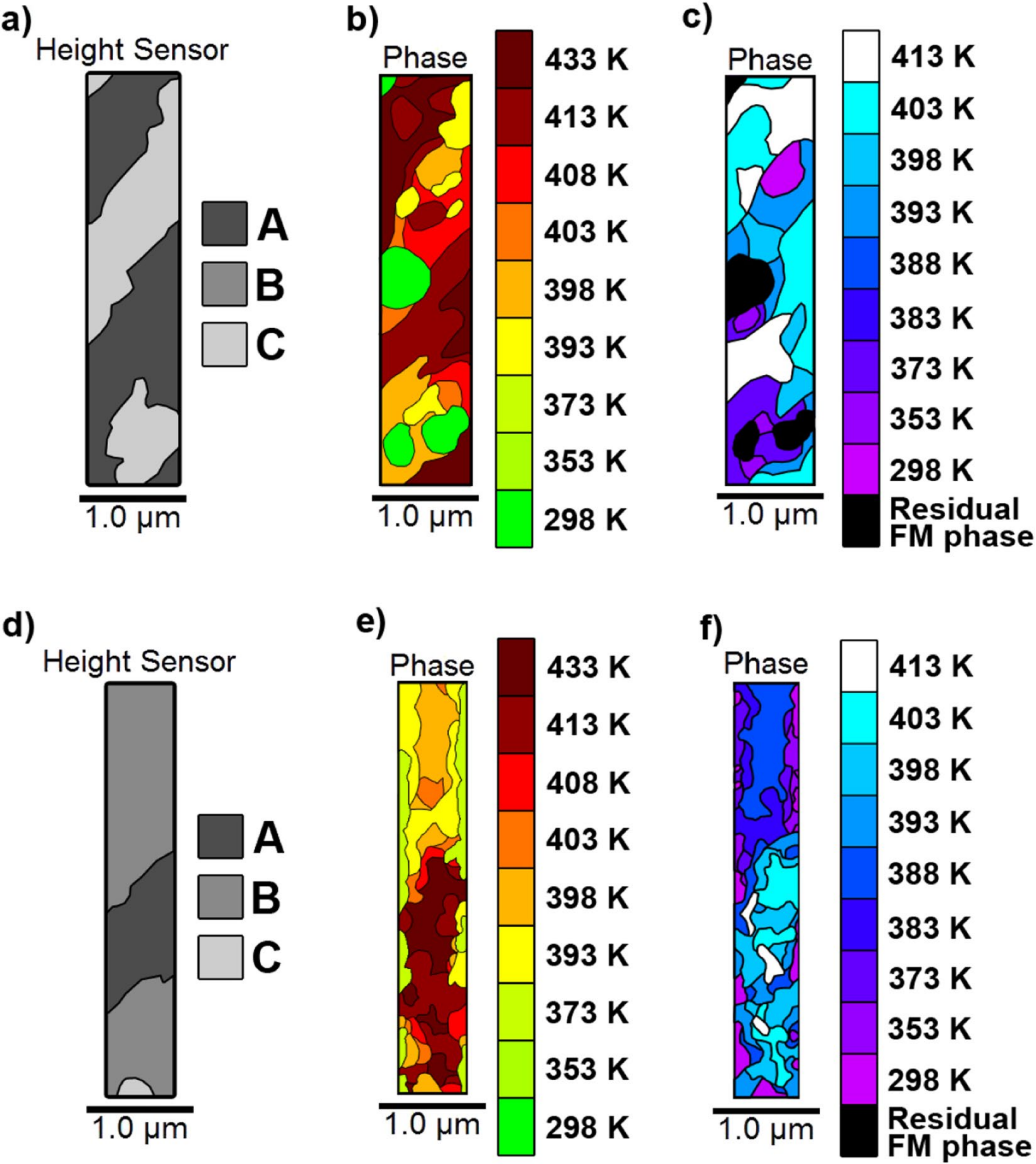
Heat maps representing the evolution of the heating and cooling transitions in 0.75 μm and 0.5 μm MgO(001)/Rh(1.1 nm)/FeRh(53 nm)/Pt(4.5 nm) nanowires. (**a**–**c**) The topography map, heating and cooling phase evolution plots respectively for the 0.75 μm wire. (**d**), (**e**,**f**) the same plots for the 0.5 μm wire. All data are plotted on the same scale to aid direct comparison.

The 0.75 µm and 0.5 µm nanowire heat maps, Fig. 8(a–c,d–f) respectively, again show strong dependence on film structure. There was a clear difference between the A-type, B-type and C-type regions, and therefore as with the 2 µm wire, the results were similar to those observed in the continuous films. Once again, the B-type structure transitioned ≈ 10 K–15 K lower than the A-type regions. As there was very little C-type structure present, there was no residual FM phase at room temperature, and little FM phase in the early stages of the heating cycle. Hence evolution of the FM phase progressed almost entirely through growth of existing domains, with little subsequent nucleation.

**Figure 9:**
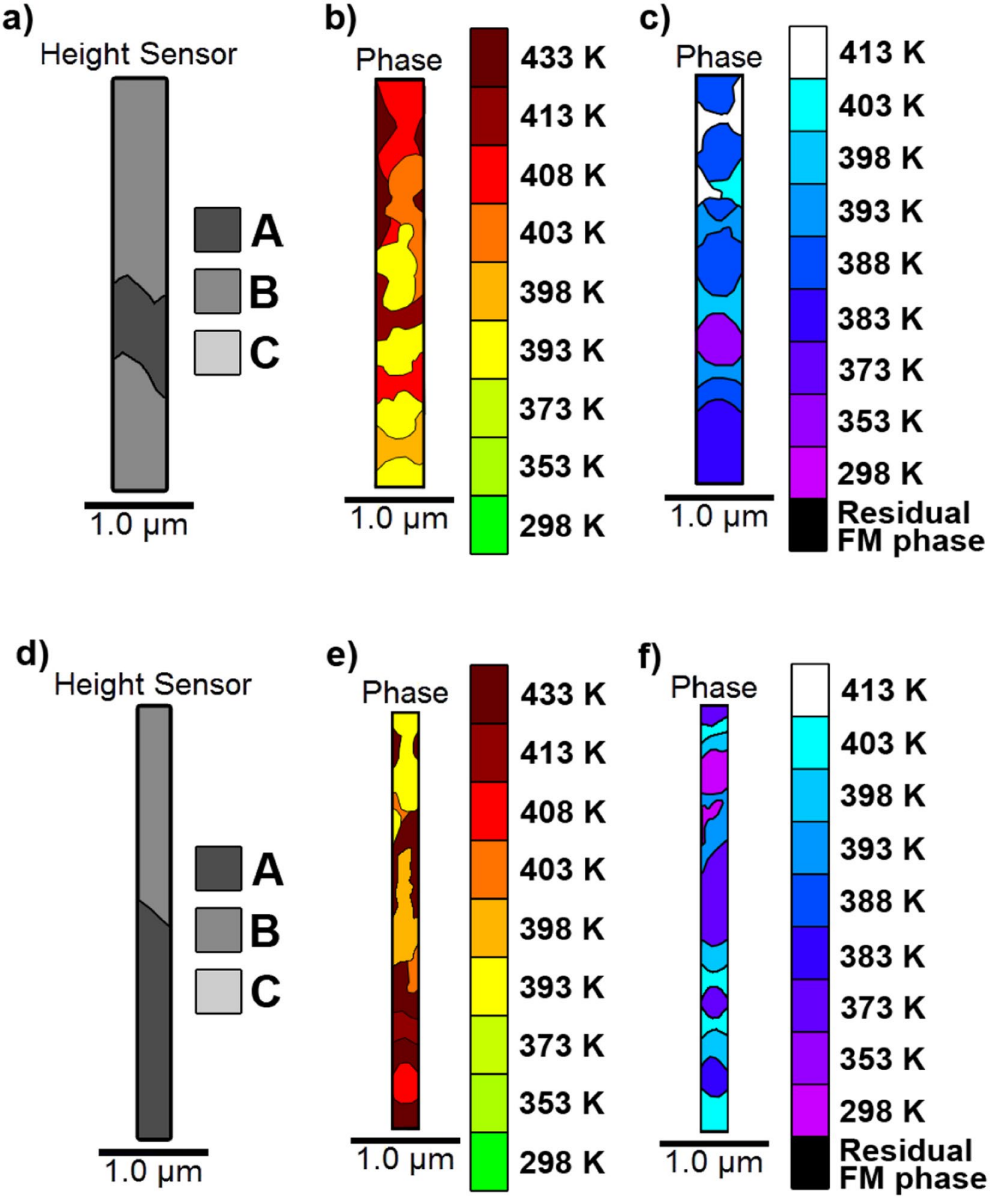
The raw and extracted data in a 0.35 and 0.2 μm wide MgO(001)/Rh(1.1 nm)/FeRh(53 nm)/Pt(4.5 nm) nanowire. (**a**–**c**) The topography map, heating and cooling phase evolution plots respectively for the 0.35 μm wire. (**d**–**f**) The same plots for the 0.2 μm wire. All data are plotted on the same scale to aid direct comparison.

The same structure-based variation in transition temperature was also observed in the 0.35 µm and 0.2 µm nanowires, Fig. 9(a–c,d–f) respectively. However, some B-type regions exhibited higher than expected transition temperatures. For both wires, their widths had been reduced to the point where they were of the same scale as regions undergoing the transition between successive temperature values (0.1 µm^2^–0.55 µm^2^). As a result, areas that had transitioned often occupied the entire width of the wire.

In the 0.5 µm wire nearly all of the initial FM nucleation occurred at the edges of the wire and spanned regions of both A-type and B-type structure. Furthermore, upon cooling the same regions were the last to transition to the AF phase. Similar behaviour was observed in the 2 µm wire, where a significant portion of the residual FM phase remaining after cooling was located along the sides. These observations indicate that a FM-favouring edge-effect resulting from either the boundary of the film or edge damage from the milling process. However the other three wires did not exhibit such a preference. The roughness and side profile of all edges were analysed, but no abnormalities in the 2 µm and 0.5 µm wires were observed. Hence it is not possible to reach a definitive conclusion on the role - if any - of edge effects in the magnetic phase transition.

## Discussion and Conclusions

The results obtained in this study clearly demonstrate that the evolution of the heating and cooling phase transitions is dominated by the variation in local film morphology. Spatially confining the FeRh films into nanowire structures produced no significant change in magnetic behaviour observed by MFM on a length scale of 50 nm. We hypothesise that the reason for the lack of dimensional sensitivity is the relative strength of film morphology effects which in our films appears much greater than effects due to the patterning process or from magnetic correlation effects. In effect, surface topography and the patterning are decoupled in terms of the magnetic transition observed in our films. These observations demonstrate that the structure and surface morphology of films and nanowires must be characterised and reported in order to fully understand and explain any results obtained.

Topological dependence of magnetic behaviour opens the possibility for tailoring transition characteristics through careful substrate preparation and film growth to modify the ratio of surface types allowing exclusion or isolation of individual morphologies. As an example, in order to study fundamental magnetic correlation lengths in is important that only one type of surface morphology is present. It also opens the possibility of creating additional film types whose identification and characterisation could reveal significantly different transition behaviour to that observed here, further improving understanding of the interplay between topography and magnetism.

The underlying reasons as to why the different regions of topography yield different magnetic behaviour remain to be understood. Compositional variations and grain boundary effects are unlikely to be responsible as there is no readily explainable physical mechanism that could be invoked. It is possible that different strain effects, possibly related to some local crystallographic variation in the substrates might provide an explanation. However, experiments to simultaneously isolate both surface topography and lattice parameter measurements with a spatial resolution of <μm represent a significant scientific challenge.

In conclusion, for the first time the metamagnetic phase transition in FeRh thin films and nanowires has been comprehensively studied through MFM imaging as a function of temperature. Surface morphology which in our films demonstrates a significant multiplicity of character has been shown to dictate the evolution of the transition, and dominate any spatial confinement effects. This highlights the need for topological characterisation of all low-dimensional samples in order to fully understand their behaviour and the fundamental nature of the FeRh transition.

## Methods

### Sample deposition and preparation

The samples were grown by dc magnetron sputtering using an 11 target AJA sputter system from a Fe_50_Rh_50_ alloy target, with a base pressure of better than 5 × 10^−9^ Torr, an Ar process gas pressure of 3 mTorr and a sputter power of 100 W. All samples were deposited on 10 ×10 mm single crystal (001) oriented MgO substrates. A substrate temperature T_Sub_ of 650 °C was used during film deposition, this temperature was then increased to 750 °C for post-deposition annealing. The films were left to cool under vacuum, until ambient conditions were established following which a 4 nm Pt capping layer was DC magnetron sputtered to inhibit oxidation. The Magnetic Force Microscopy study and X-ray diffraction measurements were done on a single sample whilst a sister sample was cut into a nominally 8 mm diameter disk using a South Bay Technology Model 360 disk cutter for VSM measurements^24^.

### Nanowire fabrication

Nanowire stripes were subtractively patterned from continuous-films which had been structurally and magnetically characterised prior to fabrication. A negative-tone electron beam resist (Micro Resist Technology ma-N 2403) was spun onto the substrate and subsequently baked at 90 °C for 1 minute giving an approximate layer thickness of 300 nm. Electron beam lithography was performed using a Carl-Zeiss Sigma system with exposure conditions tailored to the widths of the wires to be produced. An aperture of 30 μm and an accelerating voltage of 10 kV were used for all samples. The exposed resist was removed using Rohm and Hass Megaposit MF-26A developer. The resist pattern was transferred to the FeRh film using ion-beam milling in an AJA International ATC Orion Series Evaporator [269] fitted with a 4 cm Kaufman and Robinson KDC 40 DC ion source. The etch was performed with an ion source voltage and current of 400 V and 23 mA respectively and an Ar pressure of 6.1 ×10^−4^ Torr. During the mill process the sample was rotated to promote etch uniformity and minimise redeposition of milled material. The remaining resist was dissolved in MICROPOSIT Remover 1165 heated to 110 ^o^C for approximately 2 days.

### Magnetic measurements

The magnetic properties of the FeRh thin films were measured using a MicroSense model 10 vector vibrating sample magnetometer (VSM). The temperature hysteresis measurements were performed in a temperature range of 298 K–473 K with a step size of 1 K using a soak period of 30 s and a sampling average of 50. This resulted in an effect temperature sweep rate of 1.24 Kelvin/minute. A 1 kOe in-plane applied magnetic field was used to saturate the domain structure along the film plane during the measurements and signals were measured both parallel and perpendicular to this plane. Background subtraction was performed by measuring, under the same conditions, a bare MgO substrate which was then subtracted from the measurement of the film. Volume normalisation of the measured magnetic signal was performed using the nominal thickness and the disk dimensions as measured by a digital Vernier calliper. Error considerations where taken from uncertainties in the film thickness, disk radius temperature measurement and magnetic moment. The transition temperature *T*_*r*_, was estimated from a linear fit to the data close to the region where the magnetisation had reached 50% of its maximum value^24^.

### Structural analysis

XRD and XRR data were collected using a Rigaku Smartlab X-Ray diffractometer equipped with a 3 kW source producing a wavelength CuK_α1_ (λ = 1.540593(2)Å) via a Ge(220) double bounce monochromator. The step size used in these measurements was 0.01 degrees and the *2θ* range was 20–70 degrees at a rate of 0.6 degrees/minute. The FeRh lattice constant was calculated from the positions of the peaks in the XRD spectra and compared to the bulk value in order to calculate the average lattice strain and hence determine the quality of the growth. The XRR measurements were performed over the range 0–8 deg. with a step size of 0.01 deg. and a rate of 0.0078 min^−1^. The data were fitted using a Fresnel approach and Parratt algorithm via GenX reflectivity package^26^.

### Scanning probe analysis

Atomic and Magnetic force microscopy was used to investigate the surface topography and local magnetisation of continuous films and patterned nanowires. These measurements were taken on a Bruker Dimension Icon instrument in tapping mode, which allowed high spatial resolution imaging of the FeRh surface using Bruker MESP probes with a tip radius of ~35 nm. The images shown in this study were collected over a 5 × 5 μm area at a resolution of 512 × 512 pixels with a scan frequency of 1 Hz and lift height of 40 nm.

In order to directly image the FeRh phase transition temperature dependent magnetic force microscopy (MFM) studies were performed. Temperature sweeps were undertaken by placing the sample on a Bruker Dimension High Temperature Heater stage with a range of 283 K–523 K. The temperature of the stage was measured using an embedded thermocouple close to, but not precisely at, the sample position. Prior to measurement a temperature calibration was performed but this calibration does not fully account for the thermal gradient across the substrate and introduces some small additional uncertainty in sample temperature reported.

Data processing was used to flatten and subtract background planes from the data as normal for any scanning probe microscope based on a tube scanner. The corrected images for each scan were processed using Adobe Photoshop. At each temperature, the areas of FM phase were traced and filled. After correcting for slight image misalignments in scan locations the filled areas were overlaid to create the heat maps.
